# The genome resources for conservation of Indo-Pacific humpback dolphin, *Sousa chinensis*

**DOI:** 10.1038/s41597-019-0078-6

**Published:** 2019-05-22

**Authors:** Yao Ming, Jianbo Jian, Xueying Yu, Jingzhen Wang, Wenhua Liu

**Affiliations:** 1 Marine Biology Institute, Shantou University, Shantou, Guangdong 515063 P.R. China; 2 Guangxi Key Laboratory of Marine Disaster in the Beibu Gulf, Beibu Gulf University, Qinzhou, Guangxi 535011 P.R. China

**Keywords:** DNA sequencing, Genome, Marine biology, Conservation genomics, Molecular ecology

## Abstract

The Indo-Pacific humpback dolphin (*Sousa chinensis*), is a threatened marine mammal and belongs to the First Order of the National Key Protected Wild Aquatic Animals List in China. However, limited genomic information is available for studies of its population genetics and biological conservation. Here, we have assembled a genomic sequence of this species using a whole genome shotgun (WGS) sequencing strategy after a pilot low coverage genome survey. The total assembled genome size was 2.34 Gb: with a contig N50 of 67 kb and a scaffold N50 of 9 Mb (107.6-fold sequencing coverage). The *S. chinensis* genome contained 24,640 predicted protein-coding genes and had approximately 37% repeated sequences. The completeness of the genome assembly was evaluated by benchmarking universal single copy orthologous genes (BUSCOs): 94.3% of a total 4,104 expected mammalian genes were identified as complete, and 2.3% were identified as fragmented. This newly produced high-quality assembly and annotation of the genome will greatly promote the future studies of the genetic diversity, conservation and evolution.

## Abstract (summary)

**Table:** 

Design Type(s)	sequence assembly objective • sequence annotation objective
Measurement Type(s)	whole genome sequencing assay
Technology Type(s)	DNA sequencing
Factor Type(s)	
Sample Characteristic(s)	Sousa chinensis • skin of body

Machine-accessible metadata file describing the reported data (ISA-Tab format)

## Background & Summary

**Fig. 1:**
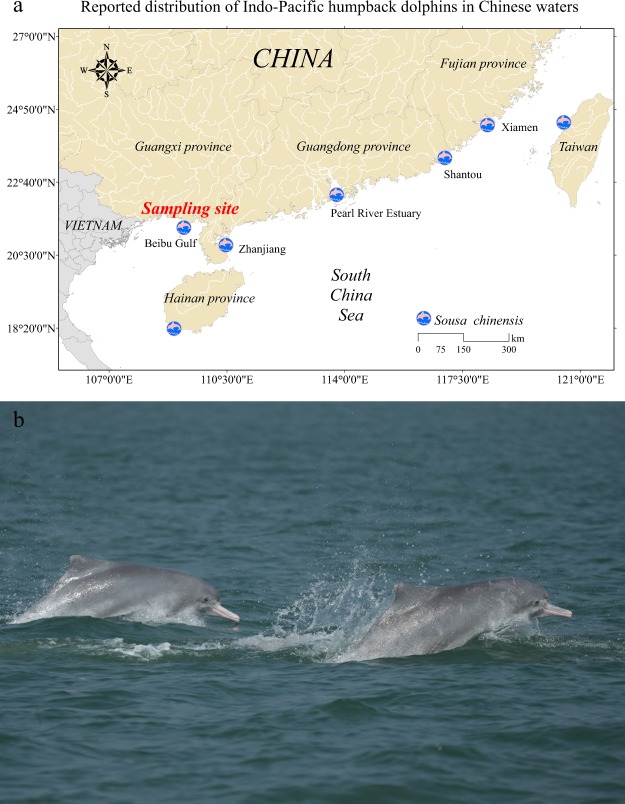
Geographical distribution and photograph of *S. chinensis*. (**a**) Distribution of *S. chinensis* reported in Chinese waters and the sampling site of this study. (**b**) *S. chinensis* photographed during the boat surveys in Guangxi Beibu Gulf, China.

The Indo-Pacific humpback dolphin (*Sousa chinensis*) normally appears in southeast Asia (in both the Indian and Pacific oceans), from at least the southeastern bay of Bengal east to central China, and then south to the Indo-Malay Archipelago^1^. The *S. chinensis* found in Chinese waters are locally known as Chinese white dolphins (the giant panda of the sea). Populations of *S. chinensis* in China have been known to be distributed from the Beibu Gulf near the border with Vietnam to the mouth of the Yangtze River^2–5^, the waters around Hainan island are also recently identified as one part of this species’ distribution^6^ (Fig. 1). At least four species are now indicated to make up the genus *Sousa*: the Atlantic humpback (*Sousa teuszii*), the Indian Ocean humpback (*Sousa plumbea*), the Australian humpback (*Sousa sahulensis*) and the Indo-Pacific humpback (*S. chinensis*) dolphins^7^. Further molecular evidence suggests that humpback dolphins in the bay of Bengal may comprise a fifth species^7^. However, as the classification and population genetics of genus *Sousa* was mainly based on the limited evidences from morphology, genetic markers and the mitochondrial sequences^7–9^, the newly produced genome of *S. chinensis* would greatly facilitate the classification and identification of *Sousa* genetic resources.

*S. chinensis* are among the most threatened cetaceans for their coastal inhabitation, which are vulnerably impacted by human activities^7^. It has been listed in the First Order of the National Key Protected Wild Aquatic Animals List in China (refer to: List of Wildlife under Special State Protection, which was designated by the Chinese State Council in 1988) and in the Convention on International Trade in Endangered Species of Wild Fauna and Flora (CITES). The species is currently categorized as Near Threatened by the International Union for Conservation of Nature (IUCN). The threats include entanglement in fishing nets (primarily gillnets), habitat destruction and degradation, vessel traffic and environmental pollutants, are all serious and fatal to *S. chinensis*^1,10–15^. As a result, much greater efforts are needed for conservation of this species to stop its apparent decline^1^. At present, most of the research has mainly focused on the morphology^16^, reproduction and growth^15,17^, population distribution^1,18^, biodiversity^19^ and toxicology studies of this species^11,20,21^. Genetic research of *S. chinensis* was mainly based on genetic markers^9^, specific genes^22^, mitochondrial DNA^8,23^ and transcriptome^24^. The genomic background and molecular mechanism of its evolution and conservation are still unknown. The high-quality whole genome sequences information would be a valuable resource for the biology, ecology, conservation and evolutionary studies.

To obtain a high-quality genome sequence of *S. chinensis*, we first performed a pilot genome survey with low depth coverage sequencing (32.9X) (Table 1) by using Illumina Hiseq 4000 to estimate the genome size and heterozygosity of the species. The assembled genome size is about 2.29 Gb^25^ (contig N50 = 13 Kb and scaffold N50 = 163 Kb) and the completed BUSCO evaluated is just about 76% in genome survey^26^. The low depth sequencing estimated the genome size is about 2.7 Gb and generated an insufficient completeness genome^26^. Therefore, we constructed four additional insert size libraries (beside the previous 500 bp and 2 Kb in genome survey) and generated a total of 290.5 Gb (107.6X) clean data after filtering (Tables 1 and 2). The *S. chinensis* genome was finally assembled into scaffolds with a total size of 2.34 Gb^27^ (Tables 1 and 3). The contig and scaffold N50 of assembly results was 67 Kb and 9 Mb, the N50 number and N90 number of scaffolds was 78 and 283 respectively (Table 3). 94.3% of 4,104 conserved genes were completed identified by BUSCO^28^ (Table 4). The newly assembled genome quality was much better than the genome survey (Table 1). In total, 878.3 Mb (37.41%) of genomic regions consist of repeat sequences (Table 5). The gene annotation of the genome yielded 24,640 coding genes and 91.2% of the predicted genome were annotated from biological databases (Tables 6 and 7). Approximately 95% of the “total complete BUSCOs” were identified by BUSCO pipeline based on the annotation result (Table 8), which suggested a good quality genome annotation.

**Table 1:** Comparison of the new genome with our previously published survey assembly of *S. chinensis* genome.

Content	The pilot study published^26^	This study
Sequencing data and depth	107.6 Gb (~32.9X clean data)	290.5 Gb (~107.6X clean data)
The number of insert size libraries	2 (500 bp and 2 Kb)	6 (300 bp, 500 bp, 800 bp, 2 Kb, 5 Kb and 10 Kb)
Genome assembly methods	SOAPdenovo2	Platanus v1.2.4
Assembled genome size	2.29 Gb	2.34 Gb
Assembled quality	contig N50:13 Kb; scaffold N50:163 Kb	contig N50: 67 Kb; scaffold N50: 9 Mb
Assembly completeness evaluation (BUSCO)	76%	94.3%

**Table 2:** Statistics of raw and clean data.

Pair-end Libraries	Insert Size	Reads Length (bp)	Raw Data (Gb)	Clean Data (Gb)	Sequence Depth (X)
	*300* bp	150	137.6	108.1	40
	*500* bp*	125	67	60.3	22.3
	*800* bp	125	59	51.2	19
	*2* kb*	50	40.7	28.5	10.6
	*5* kb	50	19	11.6	4.3
	*10* kb	50	46.9	30.8	11.4
Total			370.2	290.5	107.6

Note: Assuming the genome size is 2.7 Gb. *The data was used in previously pilot study project^26^.

**Table 3:** Statistics of the assembled sequence length.

	Contig Length (bp)	Contig Number	Scaffold Length (bp)	Scaffold Number
N10	160,909	1,135	21,984,446	9
N20	124,084	2,787	17,517,993	21
N30	100,087	4,874	14,735,920	36
N40	81,924	7,437	11,330,947	54
N50	66,998	10,567	9,008,636	78
N60	54,491	14,403	6,903,794	108
N70	42,832	19,193	5,150,637	147
N80	31,804	25,446	3,635,400	202
N90	19,905	34,515	2,124,572	283
Max length	541,590		40,839,098	
Total length	2,315,724,921	84,941	2,339,085,850	20,903

**Table 4:** Evaluation of genome assembly completeness.

BUSCO benchmark	Number	Percentage (%)
Complete BUSCOs	3,870	94.3
Complete and single-copy BUSCOs	3,802	92.6
Complete and duplicated BUSCOs	68	1.7
Fragmented BUSCOs	94	2.3
Missing BUSCOs	140	3.4
Total BUSCO groups searched	4,104	100

**Table 5:** General statistics of repeats in genome.

Type	Repeat Size	% of genome
Trf	27,926,236	1.19
Repeatmasker	592,428,741	25.23
Proteinmask	67,881,250	2.89
De novo	813,811,498	34.66
Total	878,297,072	37.41

**Table 6:** General statistics of predicted protein-coding genes (Note: The average transcript length does not contain UTR).

Gene set		Number	Average transcript length (bp)	Average CDS length (bp)	Average exon per gene	Average exon length (bp)	Average intron length (bp)
*Homolog*	*Bos taurus*	30,592	17,124	1,122	6	182	3,101
	*Tursiops truncatus*	23,909	22,700	1,315	7	180	3,398
	*Orcinus orca*	27,223	20,725	1,260	7	180	3,251
	*Balaena mysticetus*	30,618	12,062	1,025	6	180	2,360
*RNA-seq*		27,938	13,517	1,682	6	298	2,546
*Final set*		24,640	24,148	1,283	7	174	3,516

**Table 7:** Statistics of function annotation.

		Number	Percent (%)
Total		24,640	100
Annotated	InterPro	21,313	86.50
	GO	15,120	61.36
	KEGG	19,276	78.23
	Swissprot	21,734	88.21
	TrEMBL	22,235	90.24
Annotated overall		22,472	91.20
Unannotated		2,168	8.80

Note: Five protein databases were chosen to assist in predicting function of genes. They are InterPro, Gene ontology, KEGG, Swissprot and TrEMBL. The table shows numbers of genes match to each database.

**Table 8:** Evaluation of genome annotation completeness.

BUSCO benchmark	Number	Percentage (%)
Complete BUSCOs	3,900	95.1
Complete and single-copy BUSCOs	3,803	92.7
Complete and duplicated BUSCOs	97	2.4
Fragmented BUSCOs	61	1.5
Missing BUSCOs	143	3.4
Total BUSCO groups searched	4,104	100

## Methods

### Sample collection, DNA extraction and sequencing

The same sample collection and DNA extraction methods have been reported in a previously published study^26^. In addition to the previously constructed 500 bp and 2 kb libraries, new 300 bp and 800 bp small insert and 5 kb and 10 kb mate pair libraries were constructed according to the manufacturer’s protocol (Illumina, San Diego, CA, USA). After library construction, we used Illumina HiSeq X Ten to sequence PE150 reads for 300 bp library. PE125 reads for 800 bp library, and PE50 reads for 5 Kb and 10 Kb libraries were sequenced by Illumina HiSeq 4000 platform. A total of approximately 370 Gb raw data was obtained. Then, we filtered the reads with stringent filtering criteria using SOAPnuke^29^ and 290.5 Gb of clean data was generated (107.6X genome coverage) (Table 2).

### Genome assembly and evaluation

We used all the clean data to assemble the genome by Platanus^30^. First, the contigs were constructed based on the de Bruijn graphs from paired-end reads. Second, the order of the contigs was fixed using the paired end (mate-pair) information in the scaffold construction process. Third, in the Gap-closing step, each set of assembled reads were used to close the gaps, and each gap was covered with reads mapped on the scaffolds by the Platanus pipeline. After that, we filled the gaps with GapCloser^31^. Finally, scaffolds were extended by SSPACE^32^ using the mate-paired library data. The final total assembled genome length was 2.34 Gb with a contig N50 of 67 kb, and a scaffold N50 of 9 Mb (Table 3). The assembly and gene annotation qualities were assessed using BUSCO software^28^. The total number of mammal gene sets used in the evaluation was 4,104.

### Genome annotation

The genome was searched for tandem repeats using Tandem Repeats Finder^33^. Interspersed repeats were mainly identified using homology-based approaches. The Repbase^34^ (known repeats) database and a de novo repeat library generated by RepeatModeler (http://www.repeatmasker.org/RepeatModeler.html) were used. The database was mapped by using RepeatMasker (http://www.repeatmasker.org). The repeat content of this species is 37.4% (Table 5).

The coding genes in the *S. chinensis* genome were annotated based on evidence derived from known proteins and published RNA sequences. For protein homology-based prediction, proteins of *B. taurus*, *T. truncatus, O. orca*, and *B. mysticetus* were downloaded from NCBI and aligned to the *S. chinensis* genome using TBLASTN^35^ with an E-value ≦ 1E^−5^. Homologous genome sequences were aligned to the matched proteins to predict the gene models by Genewise^36^. We filtered the sequences for redundancy and retained the gene models with the highest scores. RNA-seq data provided a good supplement for gene prediction based on the homology-based method, as most of open reading frames (ORF) in the homology-based gene models were not intact. First, transcriptome data (total 4,305,634,920 nucleotides) of *S. chinensis* was downloaded from https://www.ebi.ac.uk/ena/data/search?query=ERP003522 which was sequenced by Illumina Hiseq2000 platform and published in 2013^24^. These reads were aligned to the assembled genome sequence using hisat^37^. Subsequently, hisat mapping results were merged and sorted, and transcripts were assembled using stringtie with the default parameters^38^. Finally, the Genewise results were extended using the transcripts ORFs following the strategy of the Ensembl gene annotation system^39^. This method and strategy were used extensively in the genome research^40–44^. The 24,640 (Table 6) predicted genes were then functionally annotated by aligning to five databases: InterPro^45^, Gene ontology^46^, KEGG^47^, Swissprot^48^ and TrEMBL^48^, 91.2% of the predicted genes were annotated with function (Table 7).

## Data Records

This genome assembly and annotation results have been deposited at DDBJ/ENA/GenBank^27^. Raw read files are available at NCBI Sequence Read Archive^49^.

## Technical Validation

### Evaluation the completeness of the genome assembly and annotation

To evaluate the completeness of the genome assembly and annotation, BUSCO pipeline^28^ was used to investigate the presence of highly conserved orthologous genes in the genome assembly and annotation result we obtained. BUSCO was run over the mammalian set, which includes total of 4,104 orthologue groups. 94.3% and 95.1% of the “total complete BUSCOs” were identified by BUSCO pipeline based on the genome assembly and annotation result respectively (Tables 4 and 8), which evidenced a good quality of the genome assembly and gene sets annotation.

To further evaluate the accuracy of genome, the paired-end short insert size library reads were aligned to the assembled genome by the BWA-mem (v0.7.15)^50^ with default parameters. After sorting mapped reads according to mapping coordinates in Picard (ver. 1.118) (http://broadinstitute.github.io/picard/), the mapping rate is 99.92% and the unique mapping rate is 75.81%. A total of 98.27% assembled genome was covered by the reads and the mapping coverage with at least 4X, 10X, 20X is respectively 98.16%, 97.97% and 97.32%.

### Comparison with other cetacean genomes

A total of approximately 370 Gb raw data was generated using the Illumina HiSeq X Ten and 4000 platform for the *S. chinensis* genome with 6 different kinds of insert size libraries: 300 bp, 500 bp, 800 bp, 2 Kb, 5 Kb and 10 Kb^49^. After a data filtering process, approximately 290.5 Gb of clean data, representing approximately 107.6-fold genome coverage, was obtained for genome assembly (Table 1). After being assembled by the software Platanus, the total assembled genome length was approximately 2.34 Gb with a contig N50 of 67 kb, and a scaffold N50 of 9 Mb^27^ (Table 3), which was better than the published *B. acutorostrata*, *L. vexillifer and B. mysticetus* genomes (Table 9). We predicted 24,640 coding genes in the *S. chinensis* genome (Table 6) by using a homolog and RNA-seq supplemented approach which was used extensively in the genome research^40–44^. There were 27,924 genes predicted in *O. orca* and approximately 20,000–23,000 genes predicted in the *B. mysticetus*, *L. vexillifer* and *B. acutorostrata* (Table 9).

**Table 9:** Statistics of the assembled sequence length of published cetacean genomes (*S. chinensis* included).

Species	Assembled genome size (Gb)	Genome coverage (X)	Contig N50 (Kb)	Scaffold N50 (Kb)	Number of genes	Reference
*Balaena mysticetus*	2.3	154.3	34.8	877	22,677	^51^
*Balaenoptera acutorostrata*	2.44	128	22.6	12,800	20,605	^52^
*Lipotes vexillifer*	2.53	114.6	30	2,260	22,168	^53^
*Orcinus orca*	2.37	200	70.3	12,735	27,924	^54^
*Sousa chinensis*	2.34	107.6	67	9,008	24,640	

Here, we reported the updated high-quality genome sequence of the threatened Indo-Pacific humpback dolphin. The genome resource would greatly enhance the further studies of the gene function and conservation biology of *S. chinensis*. Our study is an important step towards comprehensive understanding of the genetic background of *S. chinensis* at the genomic level. The data will be also valuable for facilitating studies of cetacean evolution, as well as population genetic and ecology.

## ISA-Tab metadata file

Download metadata file

## Data Availability

Several tools have been implemented in the data analyses, whose versions, settings and parameters are described below. (1) SOAPnuke: version 1.5.3, parameters used were -n 0.1 -l 20 -q 0.4 -d -M 1 -Q 2 -i -G–seqType 1; (2) Platanus: version 1.2.4, parameters used were: contig step: -k 32 -u 0.1 -d 0.5 -c 2 -t 30 -s 10 -m 300G; scaffold step: -t 30 –u 0.1; gapclose step: default parameters; (3) GapCloser: version 1.12, parameters used were –l 150 –p 25 –t 30; (4) SSPACE: version 1.1, default parameters; (5) BUSCO: version 3.0.2; (6) TRF: version 4.07b, default parameters; (7) Repbase: version 21.01; (8) RepeatModeler: version 1.0.4, default parameters; (9) RepeatMasker: open-4-0-6, default parameters; (10) Blast: version 2.2.26, parameters used were -F F -m 8 -p tblastn -e 1e-05 -a 5; (11) Genewise: version 2.4.1, default parameters; (12) Hisat: version 2-2.0.1-beta, parameters used were -p 4–max-intronlen 50000–sensitive–dta–dta-cufflinks–phred64–no-discordant–no-mixed; (13) Stringtie: version 1.2.2, default parameters; (14) InterPro: version 5.16–55.0; (15) GO: version 20141201; (16) KEGG: version 84.0; (17) Swissprot: version release-2017-09; (18) TrEMBL: version release-2017-09; (19) BWA-mem: version 0.7.15, default parameters; (20) Picard: version 1.118, default parameters.
